# Radical Tumor Denervation Activates Potent Local and Global Cancer Treatment

**DOI:** 10.3390/cancers15153758

**Published:** 2023-07-25

**Authors:** John D. Mitsou, Vivian Tseveleki, Foteinos-Ioannis Dimitrakopoulos, Konstantinos Konstantinidis, Haralabos Kalofonos

**Affiliations:** 1 Department of Plastic and Reconstructive Surgery, Athens Medical Center, 15125 Maroussi, Greece; 2 Laboratory of Molecular Genetics, Hellenic Pasteur Institute, 11521 Athens, Greece; tseveleki@gmail.com; 3 Molecular Oncology Laboratory, Division of Oncology, Medical School, University of Patras, 26504 Rio, Greece; fodimitrakopoulos@upatras.gr; 4 Division of Oncology, Department of Medicine, University Hospital of Patras, 26504 Rio, Greece; kalofonos@upatras.gr; 5 Department of General Robotic, Laparoscopic and Oncologic Surgery, Athens Medical Center, 15125 Maroussi, Greece; k.konstantinidis@iatriko.gr

**Keywords:** cancer neurobiology, radical denervation, tumor denervation, tumor regression, microsurgery, abscopal effect

## Abstract

**Simple Summary:**

The contribution of neuronal innervation to cancer development and persistence is not well understood. In the current study, a novel microsurgical technique for inducing radical and persistent denervation (R&P) on tumor growth was evaluated in a metastatic, solid tumor model in immunocompetent Sprague Dawley rats. Tumors were induced using the mammary adenocarcinoma cell line (HH-16.cl4). Surgical treatment resulted in tumor regression, and led to the long-term survival of the animals, extending to more than >1 year follow-up (long term survival; LTS = 87.5%), indicating the induction of a potent systemic anticancer response. In order to characterize further the anticancer response, multiple tumors were implanted on the same animal and treatment was applied to one of them. Both the treated (primary) and untreated (remote) tumor masses exhibited regression, leading to a 57.1% LTS. The R&P denervation strategy is in line with the results from the current translational research on cancer neurobiology. However, the clinical value of the approach will be verified in a pilot clinical study.

**Abstract:**

This preliminary study seeks to determine the effect of R&P denervation on tumor growth and survival in immunocompetent rats bearing an aggressive and metastatic breast solid tumor. A novel microsurgical approach was applied “in situ”, aiming to induce R&P denervation through the division of every single nerve fiber connecting the host with the primary tumor via its complete detachment and re-attachment, by resecting and reconnecting its supplying artery and vein (anastomosis). This preparation, known as microsurgical graft or flap, is radically denervated by definition, but also effectively delays or even impedes the return of innervation for a significant period of time, thus creating a critical and therapeutic time window. Mammary adenocarcinoma cells (HH-16.cl4) were injected into immunocompetent Sprague Dawley adult rats. When the tumors reached a certain volume, the subjects entered the study. The primary tumor, including a substantial amount of peritumoral tissue, was surgically isolated on a dominant artery and vein, which was resected and reconnected using a surgical microscope (orthotopic tumor auto-transplantation). Intending to simulate metastasis, two or three tumors were simultaneously implanted and only one was treated, using the surgical technique described herein. Primary tumor regression was observed in all of the microsurgically treated subjects, associated with a potent systemic anticancer effect and prolonged survival. In stark contrast, the subjects received a close to identical surgical operation; however, with the intact neurovascular connection, they did not achieve the therapeutic result. Animals bearing multiple tumors and receiving the same treatment in only one tumor exhibited regression in both the “primary” and remote- untreated tumors at a clinically significant percentage, with regression occurring in more than half of the treated subjects. A novel therapeutic approach is presented, which induces the permanent regression of primary and, notably, remote tumors, as well as, evidently, the naturally occurring metastatic lesions, at a high rate. This strategy is aligned with the impetus that comes from the current translational research data, focusing on the abrogation of the neuro–tumoral interaction as an alternative treatment strategy. More data regarding the clinical significance of this are expected to come up from a pilot clinical trial that is ongoing.

## 1. Introduction

Cancer remains the deadliest disease, affecting over 19 million people worldwide according to the WHO-International agency for research on Cancer [1]. Despite the enormous efforts undertaken to date, its morbidity and mortality remain high mainly due to the lack of control of metastatic disease, which constitutes the primary cause of cancer-related deaths [2]. Metastatic disease is considered the final frontier in cancer treatment, for which more efficacious therapies are needed [3].

Many scientists have noticed the close connection of cancer development and progression with the nervous system. The initial relevant observation can be traced back to the 2nd century BC, when the Greek physician Galen observed an increase in breast cancer incidence among melancholic compared to sanguine women [4]. Many centuries later, in the 1730s, C. D. Gendron stated that cancers are “nerve-like” and develop when nerves and the glandular tissue are mixed with lymph vessels [5]. In the 1830s, the perineural spread in a head and neck tumor was described [6]. An important discovery was made in the 1950s, when Rita Levi-Montalcini isolated the Nerve Growth factor (NGF), the first growth factor and founding member of the neurotrophins family, which was secreted in large amounts by transplanted mouse sarcoma cells. NGF was subsequently demonstrated to be uptaken by sympathetic nerve fibers and transported retrogradely to the corresponding parent neurons located in the sympathetic ganglia, causing rapid and exuberant postganglionic neurite outgrowth [7].

The question of whether neurite outgrowth could occur in cancerous tissue first arose in the 18th century, and the answer has been controversial until recently. Until the 1980s, the general view in the field of pathology was that tumors lack innervation [8]. Later on, peripheral nerve fibers, although absent inside the tumor mass, were found around human and animal tumors; J.G. Batsakis, in 1985, described the presence of large nerves located in the vicinity of human epithelial carcinomas, such as head and neck, gastric, or prostate cancers [9], and these findings have been confirmed by numerous studies that followed. A concise review on the relationship between tumor growth and innervation is presented in [10].

The possibility of therapeutic intervention in cancer by manipulating its corresponding innervation has been repeatedly proposed in the field. Almost two centuries ago (1840), this hypothesis was tested when surgeons attempted to treat tumors of the lip by transecting the trigeminal nerve and its accompanying vessels; while the transection of the nerves and vessels induced the symptomatic control of cancer, shown by reduced ulceration and pain, all four patients in the study ultimately required the surgical removal of the tumor [11]. A few studies, dating back to the 1940s, have provided evidence of the impact of denervation on cancer [12,13,14], but the results were insufficient and inconsistent. Moreover, conflicting results have been reported to show that gastrectomy combined with vagotomy in rats induces the denervation of the gastric mucosa, leading to a significant increase in the development of tumors (67%) and carcinomas (42%) [15,16], and sensory denervation was found to promote tumor metastasis [17]. These controversial findings created the impression that innervation does not have any specific impact on cancer, while autonomic nerves have almost been ignored and were considered as passive bystanders in cancer progression for a long time.

In recent years, many studies have shown a close relationship between cancer growth and progression with the presence of nerves in the tumor microenvironment, characterized as perineural invasion. As cancer progresses from preneoplastic lesions to dominant cancers, the nerve density can almost double compared to the level seen in non-neoplastic controls [18,19]. Neurons communicate using chemical signals, many of which can be recognized and leveraged by tumor cells, which in turn manipulate the neurons by sending local signals that drive the growth of neurons into the body of the tumor [20].

Moreover, inter-tumoral nerve infiltration is considered an independent factor of poor clinical prognosis in organs with high innervation density, as is the case for nearly 100% of pancreatic cancers, 80% of head and neck cancers, 75% of prostate cancers, and 33% of colorectal cancers [21]. In addition, a large number of studies presented in recent reviews have documented the close interplay of cancer with the nervous system [20,21,22,23,24,25] and point out that wound-healing and cancer share remarkable similarities with regards to the stimulatory role of nerves [23]. In contrast to the above studies, other papers do not support the dependence of cancer growth on innervation [26,27,28].

Tumor denervation studies have shown the inhibition of tumor growth, demonstrating the innervation-dependence of growing tumors. Two landmark studies in the field show that the denervation of the sympathetic nerves and the genetic deletion of the β2- and β3-adrenergic receptors prevented malignant transformation in the prostate gland and significantly slowed prostate tumor xenograft development [29]. In addition, the surgical and pharmacologic denervation of solid tumors results in the slowing or cessation of tumor growth in mice [30]. In basal cell carcinomas, denervation attenuates the early formation of tumors [31]

Several lines of evidence have suggested that β-adrenergic blockers could prevent or reduce the mortality of various cancers, such as those of the pancreas, breast, and prostate [32,33]. In prostate cancer, a phase I clinical trial showed that nerve density in the tumor was decreased by botulinum toxin injection, resulting in increased apoptosis of the prostate cancer cells [34].

In stark contrast, there are also investigations suggesting a stimulating effect of denervation on oncogenic transformation. Partial gastrectomy with denervation promotes the development of cancer-related lesions in the gastric remnant [35,36]. In addition, the vagus nerve has an antitumor effect in pancreatic cancer [16], and vagotomy accelerated pancreatic tumorigenesis and enhanced tumor growth [37]. It has also been shown that the chemical capsaicin-denervation of sensory neurons promotes mammary tumor metastasis to the lungs and heart [38].

We hypothesize that the role of the autonomic nervous system extends far beyond neurotransmission—electrical activity and blood flow regulation being rather organic, a notion that originated from the ancient Greeks, Aristotle and Galen, and later on, in the 18th century, by X. Bichat, who was the first to associate vegetative nerves with metabolism [39]. Aiming to prove or reject this hypothesis, new experimental denervated models were introduced, adopting the well-known research strategy of the “lesioning of a region and observing the effect”. During these studies, we have shown that microsurgical flaps are and do remain radically denervated for a considerable amount of time, and this condition is associated with the significant depression of wound healing and tissue homeostasis inside the radically denervated region [40,41]. In addition, a growing number of studies presented in recent reviews have documented the close interplay of cancer with the nervous system [20,21,22,23,24,25,42,43] and that regeneration and cancer share remarkable similarities with regards to the stimulatory role of nerves [23].

Based on these findings, it was thought that tumor survival and progression would probably be impossible in a field where even normal tissue homeostasis is severely compromised. In this preliminary study, we assessed the influence of R&P denervation on tumor growth and progression, as well as the survival outcome, through the abolishment of the neural–cancer cell interdependence in an aggressive metastatic breast cancer rat model.

## 2. Materials and Methods

### 2.1. Cell Line and Animal Model

The mammary adenocarcinoma cell line HH-16.cl4, characterized by aggressiveness and increased metastatic potential for distant metastases to regional lymph nodes and lungs, was obtained from the German Collection of Microorganisms and Cell lines (DSMC) (Braunschweig, Germany). Cells were grown in vitro in RPMI 1640 medium (Invitrogen, Life Technologies, Carlsbad, CA, USA) supplemented with 10% heat-inactivated FCS, in 100 cm^2^ flasks (Corning, Corning, NY, USA). When cell cultures reached 80% confluency, the cells were collected through trypsinization, using 0.5% Trypsin-EDTA without phenol red (Gibco, Billings, MT, USA).

One million (1 × 10^6^) cells, suspended in phosphate-buffered saline (PBS) buffer, were injected in immunocompetent Sprague Dawley rats (Hellenic Pasteur Institute in Athens, and Biomedical Research Foundation, Academy of Athens, Athens, Greece). Male and female Sprague Dawley rats were used at 4–9 days after birth and 50 μL of a 20 × 10^6^ cells/mL cell suspension were implanted through subcutaneous injection in the lateral dorsal area. Distinct solid tumor masses were formed, which quickly metastasized to regional lymph nodes and lungs.

Animal breeding, housing, and experiments were performed at the experimental animal unit of—Hellenic Pasteur Institute and Biomedical Research Foundation, Academy of Athens. All experimentation conformed to the EU guidelines and directives for live animal handling and experimental procedures adhered to the 3R principle. The animals were monitored daily for vital signs and a macroscopic assessment of their general condition was performed. The tumor size was assessed through measurement of the mass in the two-dimensional (length, l; width, w) axis using an electronic caliper gauge (Dyer, Lancaster, PA, USA). Estimation of the tumor volume was conducted using the following formula (l × w)/2.

### 2.2. Histochemical Analysis

Animals were sacrificed at different time intervals and the tissues were isolated, fixed, embedded in paraffin, and were processed for standard histopathological sectioning and analysis. Haematoxylin and eosin (H&E) staining was performed for enumeration and cellular morphology assessment. Tissues (lung, liver, lymph nodes) were placed in 4% paraformaldehyde upon dissection and were then embedded in paraffin. They were then processed for standard H&E staining for histopathological analysis.

### 2.3. Micro-PET/CT Scan

The rats fasted for a period of 12 h before imaging. The positron emission tomography (PET) studies were conducted using 2% isoflurane anesthesia in oxygen at 0.8 L/min. The lateral tail veins of the rats were injected with 34 MBq of 18F-FDG in a volume of 400 μL on three different days. After injection, a 60 min uptake time was allowed before 20 min of static scan per bed position. Two bed positions were employed for the whole-body scanning. During uptake time, the animal was anesthetized and its body temperature was maintained at 36 °C on the scanner bed. PET scans were performed using a Mediso scanner (Mediso nanoScan PC (Mediso, Budapest, Hungary), with 8 detector modules) with an axial field of view of 98.6 mm and a spatial resolution of 0.8 mm at the center of the scanner (with Tera-Tomo 3D PET iterative reconstruction). Before the PET scans and during the uptake time, CT scans were performed for the attenuation correction of the PET images. The X-ray beam energy set at 50 kVp, the exposure time at 300 msec, and current of 670 μA. Τhe number of projections per rotation was 480 and the slice thickness was 250 μm. For the CT image reconstruction, a modified version of the Feldkamp algorithm and the Ram-Lak filter was used. The PET and CT data were reconstructed using the Nucline software version 2.01 (Build 011.0005). The PET image reconstruction was performed using a version of the 3D OSEM algorithm (Tera-Tomo 3D PET iterative reconstruction) and voxel dimensions of 0.4 mm × 0.4 mm × 0.4 mm. All kinds of corrections (dead time, decay, scatter, attenuation, and axial sensitivity) and normalization were applied to the PET data. Image analysis was performed with the Mediso InterView Fusion software package version 3.00.039.0000 BETA.

### 2.4. Characterization of the Tumor Model

**Figure 1:**
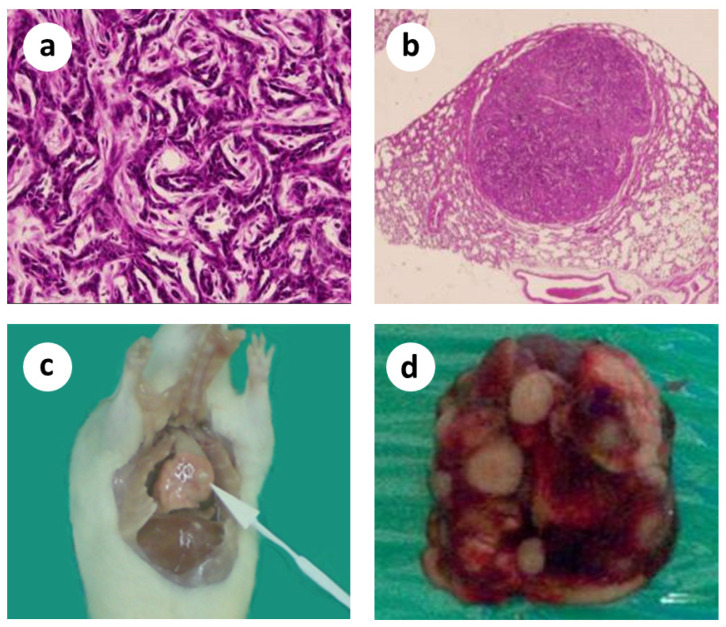
Microscopic histopathological evidence of malignancy and macroscopically visible lung metastases. (**a**) Primary tumor; (**b**) Lung metastasis; (**c**,**d**) Macroscopically visible lung metastases.

**Figure 2:**
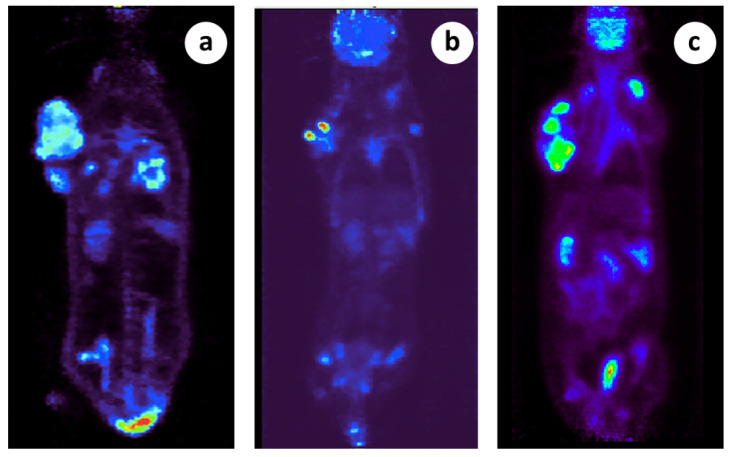
Micro PET scanning demonstration of primary and metastatic lesions in 3 different subjects. (**a**) There is a large tumor located in the right thoracic wall posteriorly demonstrating heterogeneous tracer distribution (indicative SUVmax: 3.9) and photopenic areas indicative of necrosis. Also, there is a second soft tissue mass in the right lateral chest wall close to the aforementioned large tumor likely representing a regional lymph node (SUVmax 4.2). In addition, there are multiple metastatic lung lesions bilaterally (indicative SUVmax 3.9), (**b**) There is a large tumor located in the right thoracic wall posteriorly demonstrating heterogeneous tracer distribution (indicative SUVmax: 11.2) and photopenic areas indicative of necrosis. In addition, there is a second soft tissue mass in the right lateral chest wall close to the aforementioned large tumor likely representing a regional lymph node (SUVmax 9.7). Focal FDG uptake (SUVmax 2.4) is also noted in what appears to be an additional lymph node in the left side of the thoracic cage superiorly, (**c**) There is a large tumor located in the right thoracic wall posteriorly demonstrating heterogeneous tracer distribution (indicative SUVmax: 8.8) and photopenic areas indicative of necrosis. Also, there is a second soft tissue mass in the right anterolateral chest wall close to the aforementioned large tumor likely representing a regional lymph node (SUVmax 10.0).

Before entering the study, immunocompetent rats in which subcutaneous tumors were growing were assessed through histology (Figure 1) and whole animal micro-PET scanning (Figure 2). The histopathological examination confirmed the presence of malignant tumors in all the biopsies (100%) taken from the primary tumor site. The lung biopsies taken from untreated animals at the time of surgical intervention (around 6th week) showed the presence of metastatic disease in 9 out of 17 cases (55%), which is very close to the frequency given by the cell line provider.

### 2.5. Surgical Procedure

**Figure 3:**
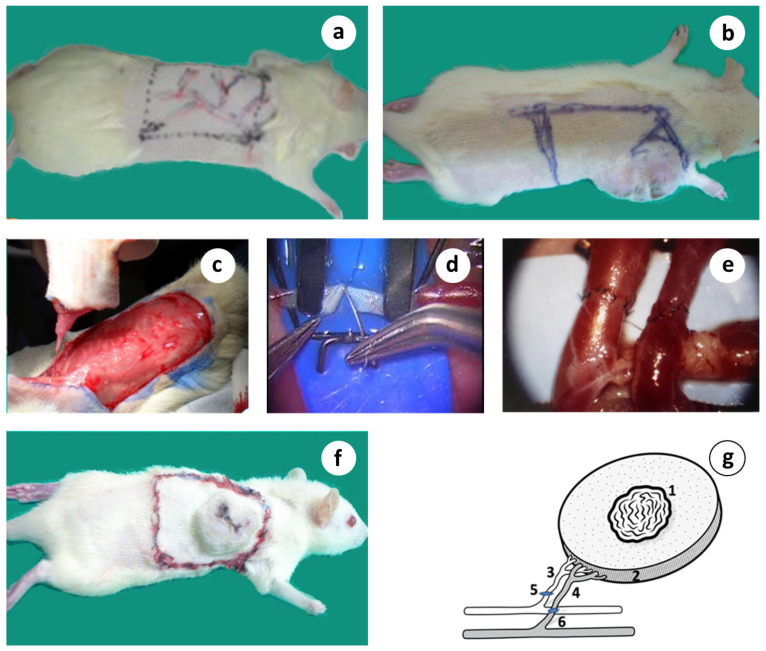
A step-by-step description of the microsurgical orthotopic tumor auto-transplantation. (**a**) Design of the proximal dorsolateral adipo-myocutaneous flap with its vascular tree marked; (**b**) Tumor about 6 wk. after induction with the tumor complex marked; (**c**) Surgical preparation of the vascular tumor complex graft isolated on its dominant vascular pedicle; (**d**) Standard hand-sewn microvascular anastomosis; (**e**) Microvascular anastomosis of both vessels completed; (**f**) Microsurgical tumor complex in place consisted by the primary tumor including a substantial amount of healthy tissue; (**g**) Schematic presentation of the microsurgical tumor composite graft based on its artery and vein; 1 = tumor, 2 = peritumoral healthy area, 3 = circumflex scapular artery, 4 = circumflex scapular vein, 5 and 6 = microvascular anastomoses.

When the tumors reached the 7–9 cm^3^ volume, about 5–7 wk. after tumor implantation, the animals entered the study. At this timepoint, most of the animals had lung or regional lymph node metastases. Anesthesia was administered with a mixture of Rompun/Imalgene, given subcutaneously. The proximal dorsolateral skin area was marked, including the tumor mass, with a substantial amount of peritumoral healthy tissue and its suppling vessels, emerging from the axillary vessels. This tumor-bearing adipo-myocutaneous preparation consisted of a large skin segment (about 7–8 × 5–6 cm), subcutaneous fat, the underlying cutaneous maximus muscle, and the dominant neurovascular bundle containing the circumflex scapular artery and vein and the accompanying nerve branch, herein called the tumor complex. This composite tumor complex was carefully dissected around and towards the axilla, where both vessels, the circumflex subscapular artery and vein, and the accompanying nerve were explored and freed from the surrounding tissues and dissected meticulously up to their origin from the axillary vessels, assisted by a Leica surgical microscope (Figure 3a–d).

Under high magnification (up to ×40), the blood vessels were transected and reconnected with microsutures (Ethilon 10/0) using the standard hand-sewn microsurgical technique (Figure 3c,d), thus restoring blood perfusion. The artery was reconnected by an end-to-end, and the vein by an end-to-side anastomosis with the axillary vein. The mean ischemia time that resulted from the duration of anastomoses was ~50–60 min. All anastomoses were performed by the same surgeon. The long-term survival (LTS) was assessed by the length of time that the animals survived without any signs or symptoms of malignancy following therapeutic intervention and were considered cured (>1 year follow up).

The unique advantage of this vascularized preparation is the cephalic dorsal position that protects the experimental area from self-attacks and auto-cannibalization. Also, the anatomy of the supplying artery and vein is quite constant and their reasonable size in rats makes them convenient for the performance of microvascular anastomosis. Despite its extent, it produces moderate trauma, as was seen by the quick recovery time of all animals. In addition, this model facilitates the handling, daily observation, and monitoring of the animals.

**Figure 4:**
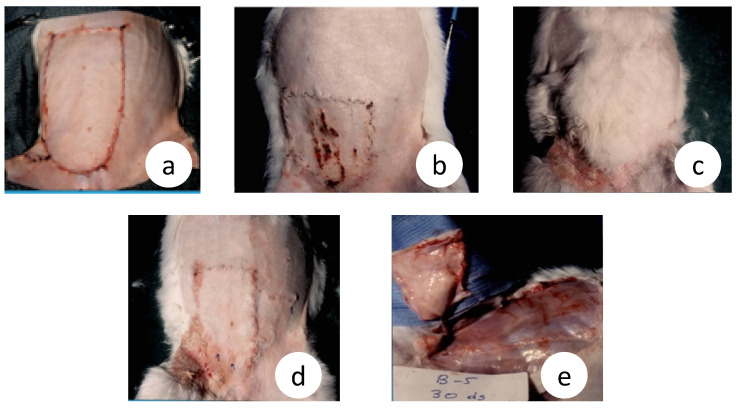
Adverse wound healing effects in microsurgical flaps and contrasting differences with the intact vascular pedicle flap, in rabbits although these 2 surgical preparations are similar regarding the blood flow dynamics. (**a**) The original size of the microsurgical and the vascular pedicle flap (day 0); (**b**) Severe contraction, trophic ulcers, and abortive hair growth inside the microsurgical flap area (day 30); (**c**) In strike contrast, vascular pedicle flap shows superabundant hair growth, light contraction without trophic ulcers (day 30); (**d**) The real size of the vascular pedicle flap after hair removal showing less contraction and comparing; (**e**) No evidence of revascularization from the underline vascular bed in a microsurgical flap which is easily separated without dissection or bleeding (day 30), (unpublished data).

The surgical procedure of the microvascular flap was initially set up in the rabbit model because, due to size, it was easier to operate upon. The results depicted in Figure 4 are from the archival records of Dr. John Mitsou and relate to the paper by Crowe et al., 1993 [40]

Whole-body, micro-PET scanning further confirmed the presence of malignancies in the primary locations and in the distant metastatic lesions, long before our surgical intervention, in all of the animals examined (*n* = 6). The metastatic load was heavy and was evident even from the 1st wk. after tumor induction (Figure 2a–c).

### 2.6. Experimental Design

The surgical technique was applied in experimental and sham operated animals. In total, 89 animals were used, assigned in 11 distinct groups, as described below.

#### 2.6.1. Group 1: Normal Control, (*n* = 6)

Tumor-bearing animals, no treatment was given.

#### 2.6.2. Group 2: Microsurgical Tumor Complex (*n* = 10)

In the microsurgical group, the tumor complex, along with its neurovascular bundle, was surgically prepared, removed from the body, and replanted, as previously described (orthotopic tumor auto-transplantation, Figure 1a–g). An ultrathin semi-permeable membrane was placed under the tumor complex to prevent any potential ingrowth of the newly formed vessels and nerve axons, allowing though the tumor-–host lymphatic exchange.

#### 2.6.3. Group 3: Vascular Tumor Complex (*n* = 10)

**Figure 5:**
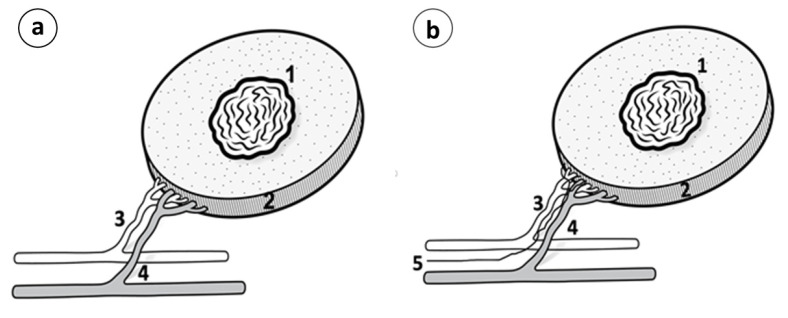
(**a**) Vascular pedicle or Vascular tumor complex; (1) Tumor; (2) Peritumoral healthy tissue and the underlying muscle; (3) Circumflex subscapular artery; (4) Circumflex subscapular vein. (**b**) Neurovascular pedicle or neurovascular tumor complex; (1) Tumor; (2) Peritumoral healthy tissue and the underlying muscle; (3) Circumflex subscapular artery; (4) Circumflex subscapular vein; (5) Accompanying nerve.

In the vascular group, the tumor complex was surgically prepared as previously, but only the nerve branch was cut, while the artery and vein were left intact (Figure 5a). The purpose was to give a good chance for tumor reinnervation through the intact vascular wall, which is a preferable neural regeneration pathway compared to other, non-neuronal tissues. A semipermeable membrane was placed under the tumor complex, as previously.

#### 2.6.4. Group 4: Neurovascular Tumor Complex (*n* = 16)

In the neurovascular group, the tumor complex was surgically prepared, but the entire neurovascular bundle was left intact (artery, vein, and nerve, Figure 5b), attempting to further increase the chance for reinnervation via the intact nerve branch compared to the previous group 3. A semipermeable membrane was placed under the tumor complex, as previously.

#### 2.6.5. Group 5: Complete Destruction of the Natural Tissue Continuity in 2-Steps, without Microvascular Anastomoses (*n* = 10)

In this group, complete detachment of the tumor complex was achieved in 2 surgical steps without microvascular anastomoses execution. In the first step, the vascular tumor complex was routinely prepared as in vascular group 3 (Figure 5a). After 10–14 days, a time that was found to be adequate for the tumor complex to gain blood flow independence from its vascular pedicle, the latter was ligated and divided. This experiment was intended to investigate the differential influence of the natural tissue continuity, per se, between the tumor complex and the host versus the microvascular anastomosis.

A semipermeable membrane barrier was also placed under the tumor complex.

#### 2.6.6. Group 6: Microsurgical Tumor Complex, Membrane Sham Control Group (*n* = 6)

In this group, the microsurgical tumor complex was created as in group 2, but the membrane placement was omitted, intending to identify any possible distinct effect of the marginal revascularization/reinnervation process emerging from the underlying vascular bed compared to the membrane-bearing control (group 2).

#### 2.6.7. Group 7: Vascular Tumor Complex, Membrane Sham Control Group, (*n* = 6)

The vascular tumor complex was created as in group 3, but the membrane placement was omitted, intending to identify any possible distinct effect on survival derived from the marginal revascularization/reinnervation process emerging from the underlying vascular bed compared to the membrane-bearing control (group 3).

#### 2.6.8. Group 8: 2-Step Destruction of Natural Tissue Continuity, Membrane Sham Control Group (*n* = 6)

Destruction of the naturally established host–tumor continuity was performed in 2 successive surgical steps, as in group 5. In group 8, membrane placement was omitted, intending to identify any possible distinct effect on survival due to the marginal revascularization/reinnervation process emerging from the underlying vascular bed compared to its control (group 5).

#### 2.6.9. Group 9: Tumor Complex Reperfusion Ischemia Sham Control (*n* = 6)

The vascular tumor complex was surgically prepared as in group 3, and the blood vessels were clumped for 50 min using a microvascular clamp (FST), which was the mean time of ischemia needed to perform the microvascular arterial and venous anastomoses. With this experiment, it was intended to identify any possible distinct effect on survival derived from the potential tissue ischemia-reperfusion injury induced inside the tumor complex during the performance of microvascular anastomoses.

#### 2.6.10. Group 10: Microsurgical Tumor Complex with Multiple Tumors (*n* = 7)

Aiming to demonstrate the apparent systemic therapeutic effect observed in the microsurgical tumor complex group carrying only 1 tumor, a multiple tumor experiment was designed. Two or three tumors were simultaneously induced on the same subject, the first one in the usual frontal dorsolateral position, and the second and third remote tumors in the backside and frontal contralateral ones, respectively. Microsurgical tumor complex was performed in the former tumor only, while the remote tumors were left untreated. A semipermeable membrane was placed under the primary tumor complex.

#### 2.6.11. Group 11: Neurovascular Tumor Complex with Multiple Tumors (*n* = 6)

Surgical preparation was performed as in Group 10, but the entire neurovascular bundle (artery, vein, and nerve) was left intact. A semipermeable membrane was placed under the tumor complex.

### 2.7. Statistical Analysis

Statistical analysis was performed using Statistical Package for Social Sciences version 17 (SPSS, Chicago, IL, USA). T test was used for paired comparisons between continuous variables with normal distribution. *p* < 0.05 was considered statistically significant for all comparisons.

## 3. Results

### 3.1. Radical and Persistent Denervation Constantly Leads to Primary Tumor Regression

When the animals received no treatment (control group 1), the primary tumors grew rapidly and the animals died or were sacrificed for ethical reasons due to the large tumor size or due to their poor general condition; this usually occurred between 9–12 wk. after tumor induction.

**Figure 6:**
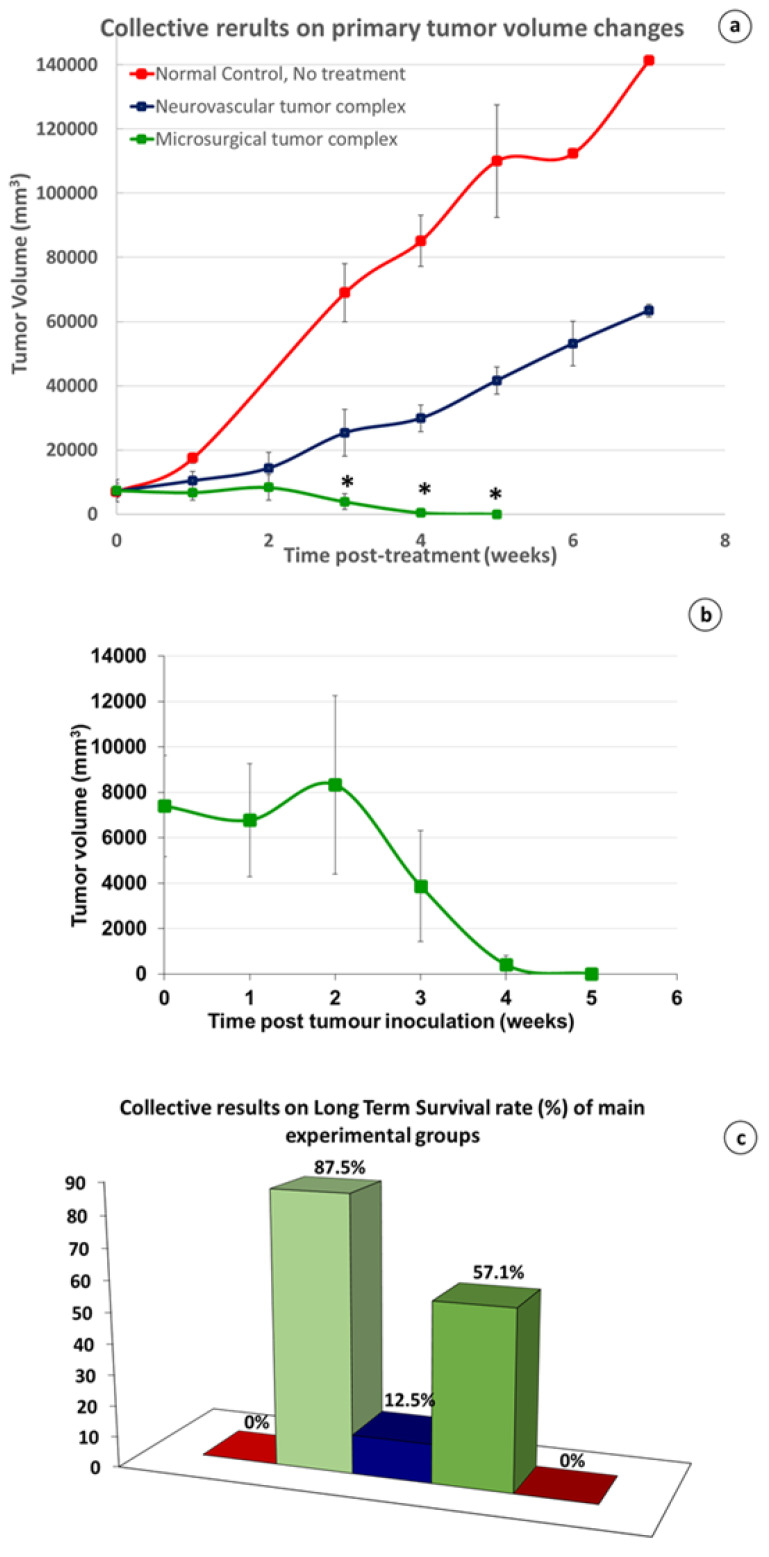
(**a**) Collective, comparative results on primary tumor volume changes. Redline, no treatment normal control (group 1); An almost linear, and sharp increase in the tumor size. Blueline, partial denervation, neurovascular groups with or without membrane (group 4 and 7): A similar type but with a moderate increase was shown. Greenline, radical and persistent (R&P) denervation, microsurgical groups with or without membrane (group 2 and 6); After an initial, light increase during the first 2 wk. after treatment, tumor deteriorates gradually until its complete and irreversible regression in microsurgical group 2 (*: *p* value < 0.005 in comparison of microsurgical tumor complex group with both control and neurovascular tumor complex groups). (**b**) Analytical presentation of the primary tumor volume changes in microsurgical group 2; after an initial moderate growth for about 2 wk. the tumors started to constantly decrease in size until their elimination, in about 5–7 wk. after treatment. (**c**) Collective results on LTS rate: from left to right: 0% = Normal control group, no treatment (group 1); 87.5% = microsurgical groups, R&P denervation (groups 2 and 6), 12.5% = neurovascular groups, partial denervation (groups 4 and 7); 57.1% = microsurgical group with 2 or 3 tumors (group 10); 0% = 2-step destruction of tissue continuity, with or without membrane (groups 5 and 8).

In microsurgical group 2, after an initial limited growth for about 2 wk. after the surgical intervention, the tumors started to constantly decrease in size until their complete elimination about 5–7 wk. after the treatment (Figure 6a,b). The pattern of the primary tumor regression was similar: tumor growth from the periphery (tumor stroma) evidently stopped, preventing further invasion and spreading, while the healing process occurred in the central “necrotic” area. It was macroscopically visible that tumor mass shrinkage occurred from the outer or stromal region to the necrotic core.

**Figure 7:**
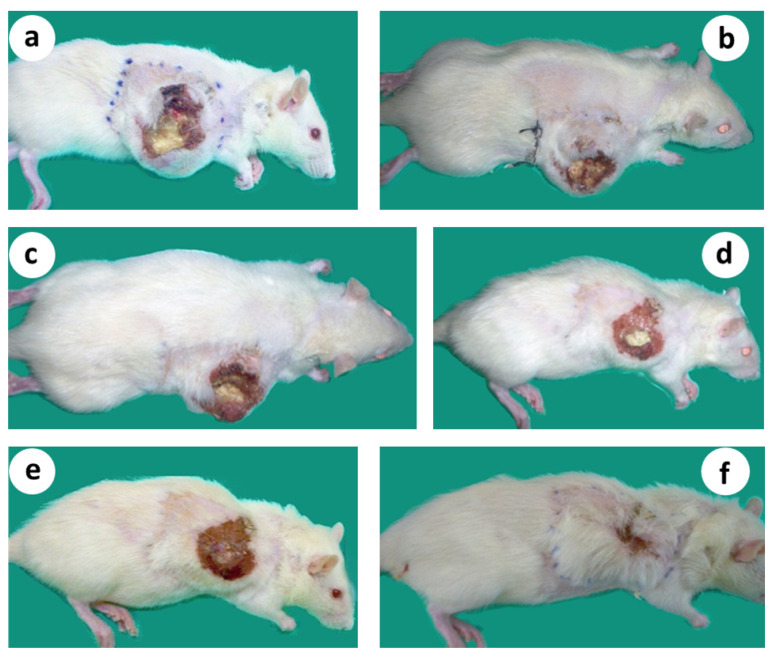
Gradual shrinkage of the transplanted tumor, followed by complete tumor regression in about 6 wk. after treatment, leading to relapse-free survival for more than 12 months (follow up period). (**a**) Microsurgical tumor complex 2 wk. after R&P denervation; (**b**–**d**) Steady reduction in the size of the primary tumor; (**e**,**f**). Primary tumor regressed following a classical wound-healing contraction pattern.

Finally, the central “necrotic” area healed completely, following the classical contraction pattern of wound healing (Figure 7a–f). No local recurrence was observed, and the animals were in good general condition, increasing their body weight steadily. In neurovascular group 4, where the neurovascular bundle was left intact, the size of the tumor of the non-cured subjects increased moderately during the first 2 wk. after treatment, but later the growth rate steadily increased at an exponential rate (Figure 6a).

### 3.2. High Rate of LTS following Primary Tumor Regression

When the animals received no treatment (Control group 1), the LTS rate was 0% and the mean survival (MS) time was 10.3 wk.

In microsurgical group 2, nine of the ten animals survived and the LTS rate reached the level of 90%, and the MS time was 46.6 wk., both being significantly higher (*p* < 0.001, student’s *t*-test) than normal control group 1 (Figure 7c,d). The non-cured animal died about 2–3 wk. after the surgical intervention due to its poor general condition, although its tumor was shrinking. Micro-PET scanning documented a heavy metastatic load in six out of the six examined subjects as early as from the first week post-tumor induction (Figure 2). Therefore, our speculation is that the animal that did not survive was already at an overly progressed disease stage for treatment to be effective.

**Figure 8:**
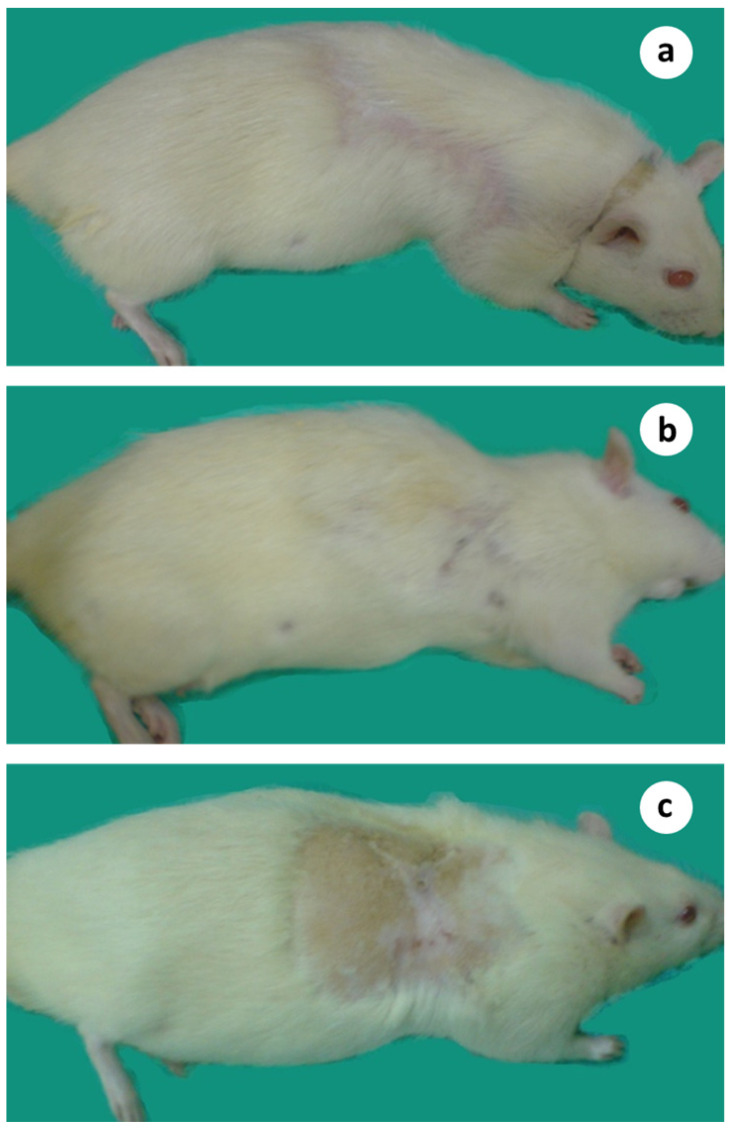
Animals survived more than one year after microsurgical tumor auto-transplantation. (**a**) 13 months; (**b**) 15 months; (**c**) 18 months after treatment.

The subjects that survived after the surgical treatment from various groups were followed up for more than a year, having no symptoms or signs of the disease (Figure 8a–c). No toxicity of any kind was observed during that period.

### 3.3. The Crucial Role of Microvascular Anastomosis

**Figure 9:**
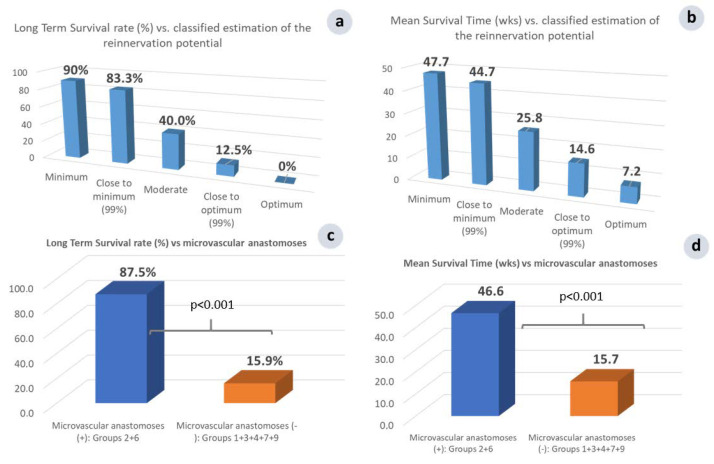
Comparison of LTS rate (**a**) and MS time (**b**) vs. the classified relative estimation of the reinnervation potential uncovered an inverse relationship of the therapeutic response to the reinnervation potential. Minimum (group 2) = microsurgical group with membrane; Close to minimum (group 6) = microsurgical group without membrane; Moderate (group 3) = vascular group; Close to optimum (group 4) = neurovascular group with membrane; Optimum (group 7) = neurovascular group without membrane. Overall LTS rate (**c**) and MS time (**d**) vs. microvascular anastomosis performance; Groups 2, 6 = microsurgical tumor complex with or without membrane, Groups 1, 3, 4, 7, 9 = Normal control, Vascular group, Neurovascular group with or without membrane, and Ischemia sham control group.

Compared to all of the other groups, microsurgical group 2 showed superiority in all of the recorded parameters; the size of the tumor had the most intense declining course (Figure 6a,b). The LTS rate and MS time were the highest recorded in the study, with (90% and 47.7 wk., respectively) or without a membrane barrier (83.3% and 44.7 wk., respectively) (Figure 9a,b), and all of those values were statistically significant (*p* < 0.001) compared to normal control group 1 (0% and 10.3 wk., respectively). The importance of the microvascular anastomoses was demonstrated by comparing the data in all of the treated animals, with or without microvascular anastomoses; the LTS rate was 87.5% vs. 15.9% and MS time 46.6 vs. 15.7 wk., respectively, both being statistically significant (*p* < 0.001) (Figure 9c,d). Statistical analysis among various groups with or without microvascular anastomoses in 64 subjects unveiled the role and contribution of each operation protocol parameter to the results obtained.

Investigating the outcome based on the facilitation of the post-operative tumor reinnervation, two additional groups of animals were implemented, as follows: The vascular tumor complex (group 3), where the artery and vein were left intact, thus permitting tumor reinnervation through the intact artery and vein, while the nerve was severed; and the neurovascular tumor complex, where the accompanying nerve was additionally preserved (group 4), enhancing even more the possibility of the reinnervation of the cancerous tumor.

**Figure 10:**
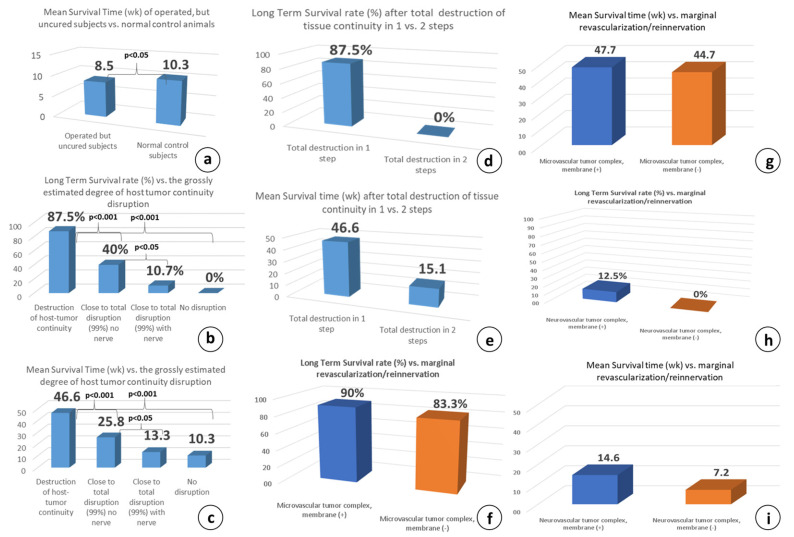
(**a**) Cumulative results of the MS time in all operated but non-cured subjects has shown that it is significantly less (*p* < 0.05) compared to the untreated, normal control animals; (**b**) LTS rate and MS time (**c**) correlation after destruction of host–tumor complex tissue continuity: Destruction of host-tumor continuity (groups 2, 6) = microsurgical group with or without membrane, Close to total destruction, no nerve (group 3) = vascular group, Close to total destruction, with nerve (groups 4, 7, 9) = neurovascular group with or without membrane, and no destruction at all (group 1) = Normal control group. LTS rate (**d**) MS time and (**e**) correlation after the destruction of host–tumor complex tissue continuity in 1 versus 2 surgical steps shows that destruction in 2 steps eliminates the possibility for long-term survival, significantly suppresses the MST time: Destruction of tissue continuity in 1 step = microsurgical group with or without membrane (groups 2, 6), destruction in 2 steps, with or without membrane (groups 5, 6). Comparison of survival of the microsurgical (**f**,**g**) and neurovascular groups (**h**,**i**) in correlation to membrane insertion, shows no significant differences in both, LTS rate and MS time: Microsurgical group with or without membrane (groups 2, 6), Neurovascular group with or without membrane (groups 4, 7).

Compared to microsurgical group 2, the LTS rate in vascular group 3 sharply decreased, from 90% to 40% (Figure 9a), and the MS time from 47.7 to 25.8 wk. (Figure 9b). The additional preservation of the main nerve innervating the skin-flap in neurovascular group 3 significantly (*p* < 0.05) further increased the morbidity and mortality; the LTS rate was 10.7% vs. 40% and the MS time was 13.3 vs. 25.8 wk. (Figure 10b,c). An almost linear, inversely proportional relationship between the reinnervation potential and survival rate has been recorded. This is a landmark finding, which further supports our approach, showing that R&P denervation was probably the key effector of the observed local and systemic anti-cancer effect.

It is worth mentioning that the influence of the R&P denervation on the fate of the tumor-bearing animals seems to be an “all or none” phenomenon, and this statement is supported by the comparison of the MS time in all of the operated, but non-cured, subjects. Whenever either R&P or partial denervation failed to induce long-term survival, the MS time was significantly less (*p* < 0.05) compared to control group 1 (8.5 vs. 10.3 wk., Figure 10a). In other words, partial denervation causes the worsening of the animals earlier than in control group 1, where the subjects did not receive any treatment.

### 3.4. The Importance of Natural Tissue Continuity between the Host and Tumor Complex

The destruction of tissue continuity is generally considered to be the loss of the tissue architecture and the abolishment of the structural boundaries, called basement membranes, which separate the epithelium from the connective tissue. The significance of preserving tissue continuity, which greatly facilitates the reinnervation process through the powerful phenomenon of collateral sprouting, has been tested with vascular group 3, where the host and tumor complex is connected by only the dominant artery and vein, without any nerve branch. Compared to microsurgical group 2 and despite the preservation of the minimally possible tissue continuity with the complete absence of neural input, the survival differences were significant, at LTS rates of 10.7% and 87.5% (*p* < 0.001, Figure 10b) and MS times of 13.3 and 46.6 wk. (*p* < 0.001, Figure 10c), respectively.

Consequently, the destruction of the host–tumor complex continuity as an independent factor was effective only when it included the nerve branch and the anatomical elements to which the regenerating axons show affinity and tropism, e.g., the blood vessel wall. The significant decrease in survival following the omission of the tiny vascular pedicle dissection indicates a possible association of tumor-promoting factors not only with the nerve branches, but also with the neural axons running along the blood-vessel wall. This may also explain the failure of the therapeutic effect of the cancerous tumors when only the corresponding nerve branches were transected, which has been attempted many times between 1840 and today [11]. Therefore, microvascular anastomoses stands out as the key therapeutic step in the proposed surgical protocol.

Unexpectedly, when the complete abolishment of the host–tumor complex continuity was performed in two successive surgical steps, spaced 10–14 days apart (group 5), the systemic anticancer effect seen in the one-step microsurgical group 2 was completely abolished and all of the animals died (LTS 0% vs. 87.5%, Figure 10d,e), although the majority of tumors showed significant or complete local remission. Curiously, this regression was temporal, followed by tumor regrowth, and the most impressive finding was the re-appearance of some primary tumors that were previously macroscopically absent. Nevertheless, the MS time improved, being significantly higher than control group 1 (19.1 vs. 10.3 wk., *p* < 0.05).

The difference between these two surgical preparations is critical as far as the potential for marginal revascularization/reinnervation is concerned because in the microsurgical tumor complex, the blood flow is immediately restored and remains high thereafter, thus preventing the potential marginal revascularization/reinnervation process inside the tumor. Instead, when the complete destruction of tissue continuity is performed in two successive surgical steps, the vascular pedicle serving the tumor complex is suddenly transected after 2 wk., inducing abrupt ischemia inside the tumor complex. This eventually activates compensatory marginal revascularization, which will inevitably be combined with a subsequent reinnervation process (discussed later).

### 3.5. The Effect of Marginal Revascularization/Reinnervation Process

An important issue during the planning of the present study was the possible implication of the marginal revascularization/reinnervation process. To answer this question, two sham-operated control groups (groups 6 and 7) were included, in which membrane placement was omitted, intending to identify any possible effect of the marginal revascularization/reinnervation process emerging from the underlying vascular bed compared to the membrane-bearing counterparts, microsurgical group 2 and vascular groups 3. A sham operation is a procedure that is similar to the main therapeutic approach, but with the omission of the key therapeutic element under investigation, and is an important scientific control.

When the membrane was omitted in microsurgical group 6, five out of the six subjects were cured; the LTS rate was 83.3% and the MS time was 44.7 wk., both being comparable to those of the membrane-bearing subjects: 83.3 vs. 90% and 47.7 vs. 44.7 wk., respectively (Figure 10f,g). This indicated that the underlying marginal revascularization/reinnervation probably has no significant contribution to the therapeutic outcome, and no reinnervation occurs from the underlying vascular bed. Similarly, in the neurovascular membrane sham control group 7, six out of the six subjects were euthanatized due to tumor progression. The LTS rate was 0% and the MS time was 7.2 wk., both being equivalent to the neurovascular counterpart group without membrane (0% vs. 12.5% and 7.2 vs. 14.6 wk.), supporting again that the underlying marginal revascularization/reinnervation probably has no significant contribution to the therapeutic effect (Figure 10h,i).

### 3.6. Possible Role of Surgical Traumatic Severity on Survival

**Figure 11:**
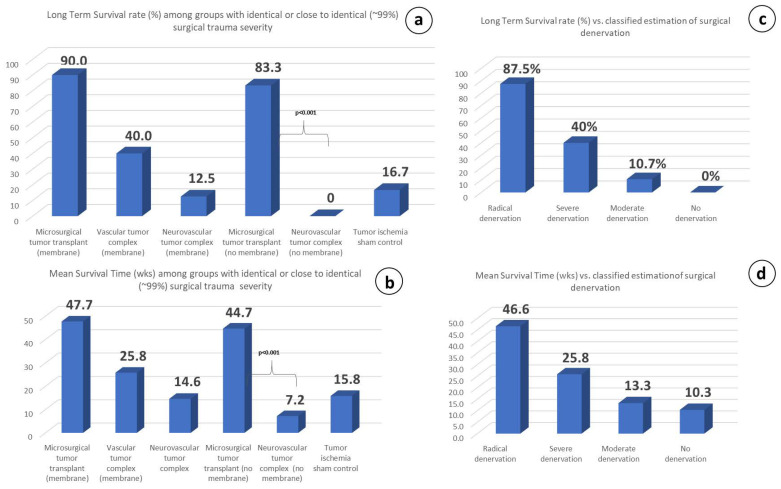
LTS rate (**a**) and MS time (**b**) vs. surgical trauma severity. Animal groups with identical or close to identical trauma have shown wide variations on both parameters, indicating no direct influence of trauma severity on the results: Microsurgical tumor complex with membrane (group 2), Vascular group without membrane (group 4), Neurovascular group with membrane (group 4). Microsurgical tumor complex without membrane (group 6), Neurovascular group without membrane (group 7), Tumor ischemia sham-control (group 9). LTS rate (**c**) and MS time (**d**) vs. classified, relative estimation. A proportional relationship of the therapeutic response to the degree of the promptly induced surgical denervation has been recorded. R&P denervation (groups 2, 6) = Microsurgical tumor complex with or without membrane; Partial but severe denervation (group 3) = vascular tumor complex, Partial moderate denervation (groups 4, 7) = neurovascular tumor complex with or without membrane; No denervation (group 1) = Normal control.

To address this issue, we compared the results between the groups with identical or very close to identical (~99%) severity of trauma; i.e., the microsurgical, vascular, and neurovascular tumor complex with or without membrane, where 64 subjects were assigned to five different groups (2, 3, 4, 6, and 7), across whom wide variations of the LTS rate and the MS time were recorded (Figure 11a,b).

Characteristically, microsurgical group 2, having a tiny difference in the traumatic severity (about 1%) compared to neurovascular group 4, has shown exceptional differences (*p* < 0.001) concerning the therapeutic response in both the LTS rate (83.3% vs. 0%) and MS time (44.7 vs. 7.2 wk.), with the only difference between these two groups being the preservation or lack of preservation of the neurovascular pedicle tissue-continuity.

### 3.7. Partial vs. Radical and Persistent Denervation

It was shown in our experiments that partial denervation of any kind may have even accelerate disease progression in the experimental animals compared to the non-treated control group 1 (Figure 9c). In contrast, whenever the microsurgical tumor complex was performed, the primary tumors always regressed (Figure 6a,c). The comparison of the therapeutic response with the degree of surgically induced denervation has revealed that the therapeutic effect was proportional to the degree of denervation; the greater the extent of denervation, the stronger the therapeutic response, as recorded for the LTS rate and the MS time in each group (Figure 11c,d).

On the other hand, summarizing the results of the therapeutic potential vs. the classified grossly estimated reinnervation potential, it seems that a disproportionate relationship governs both the LTS rate and the total MS time in each group; i.e., the lower the reinnervation potential, the higher the possibility for a definite cure (Figure 9a), and the longer the MS time, and vice versa (Figure 9b).

### 3.8. Multiple Tumor Experiment: Regression of the Remote, Non-Operated Tumors

**Figure 12:**
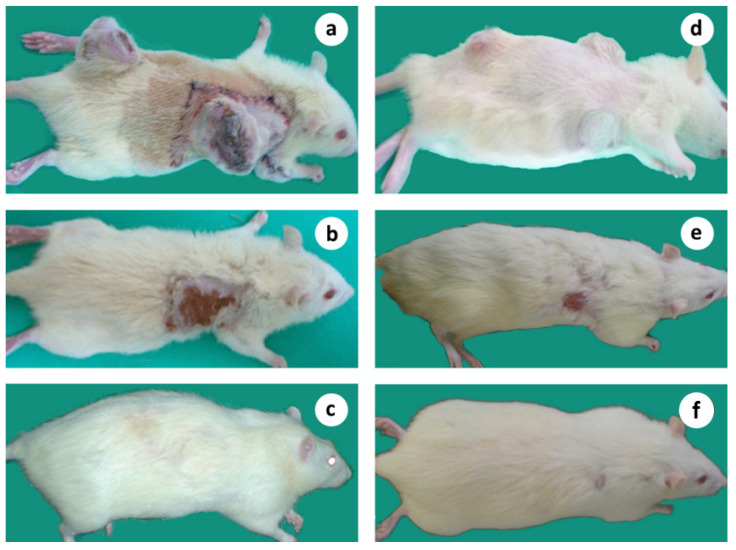
Microsurgical tumor complex in a subject bearing 2, simultaneously induced tumors; (**a**) Animal with 2 tumors 7–8 wk. after tumor induction, on the day of treatment, applied to only one of the tumors; (**b**) The same animal showing the regression of both tumors, 10 wk. after treatment; (**c**) The cured animal 14 months after tumor induction without relapse of malignancy. (**d**–**f**) Microsurgical tumor complex in a subject bearing 3, simultaneously induced tumors; (**d**) Animal with 3 tumors, 5 wk. after tumor induction; (**e**) The same animal showing the regression of all tumors 13 wk. after treatment; (**f**) The cured animal 12 months after tumor induction without relapse of malignancy.

In the microsurgical group bearing multiple tumors (group 10), in four out of the seven animals, both the operated and remote untreated tumors gradually regressed, and eventually disappeared about 5–8 wk. after treatment with no local or systemic disease recurrence, and the animals stayed in a good general condition thereafter (Figure 12a–f). The LTS rate of 57.1% was significantly higher (*p* < 0.05) compared to normal control group 1 (57.1% vs. 0%), but was similar to microsurgical group 2 bearing only one tumor (group 2). The MS time of the treated, but non-cured, subjects was only 7.4 wk., which was close to control group 1 (7.4 versus 10.3 wk.).

In the neurovascular group bearing multiple tumors (group 11), in which the blood vessels and the accompanying nerve were left intact, in one out of six subjects, the treated ‘primary’ tumor regressed, followed by the concomitant regression of the ‘remote’ one, leading to its long-term survival (LTS = 16.7%). This result did not differ significantly from normal control group 1. The MS time of the treated but non-cured subjects was 7.2 wk., which, again, was equivalent to the untreated control group 1 (7.2 vs. 10.3 wk.).

No systemic toxicity of any kind was noticed in either group. Overall, the surgical procedure was well tolerated, as it did not induce extensive trauma and it did not involve internal organs. Recovery was very quick after the surgery.

## 4. Discussion

In recent years, an ongoing number of studies have begun to shed light on the role of the nervous system in cancer initiation and progression, and a new field of cancer neurobiology is emerging. However, the knowledge has not yet been translated into effective therapeutic interventions. In this study, using an aggressive and metastatic rat breast tumor model, we show that R&P tumor denervation may lead to local and global cancer treatment, without primary tumor removal.

It must be emphasized that no toxicity of any kind has been noticed and, if clinically proven, it can be utilized without restrictions in many categories of patients, such as those with serious underlying diseases, patients with advanced cancer disease, or even patients in need of fertility-sparing treatment [44], etc.

If the radical and persistent separation of the tumor cells from the autonomic and sensory nerves leads to the death of the former, an essential question arises: How do the terminally differentiated neurons, being in a post-mitotic state, manage to interact so strongly with the prokaryotic-like cancer cells, despite their immense differentiation state disparity?

Human morphological data confirmed that perineural invasion promotes a symbiotic relationship via an ultimate and active interaction between cancer cells and nerve fibers [45]. Striking phenotypic similarities between wound healing and cancer have been noted by many authors over time [46,47,48], and it has been proposed that wound healing in cancer is abnormal [49] or misregulated [50]. Therefore, to understand the implication of the peripheral nerves in tumor biology, it is helpful to consider the profound and dynamic changes that take place during the healing process, thus building their strong interdependence.

Healing is a timed, strictly ordered, and hierarchical process, mirroring the critical steps of the evolutionary and developmental/ontogenetic path in a bi-directional way. The initial profound phenotypic de-differentiation of the involved tissues [51] is followed by a stepwise re-differentiation, leading to the complete restoration of the original structure.

The wound healing process necessitates the destruction of the evolutionary/ontogenically established natural tissue compartmentalization provided by the basement membranes; therefore, wounding presents a deliberate thermodynamic challenge [52]. This could further explain the absence of complete regeneration in highly evolved organisms, like humans, in which healing is rapid, but incomplete. It seems that nature sacrifices perfection for the sake of survival.

This statement could also explain the apparent reciprocal relationship among the regeneration/healing capacity and tumorigenicity during evolution, where the former is decreased, while tumorigenicity is increased, in evolved organisms. Indeed, neoplasia incidence in invertebrates is less common than in vertebrates, and in some invertebrate taxa, tumors are virtually absent and most of them are benign [53]. Also, the reported lifetime risk of developing cancer in humans is around 40% [54], whereas in mammals it is 3%, in birds 2%, and in reptiles 2% [55].

The evolutionary and developmental profile of wound healing has been highlighted by other investigators [56,57,58]. Interestingly, it was recently shown that carcinogenesis may represent a paradigm of reverse evolution from multicellular organisms to unicellular ones, explaining the metastatic ability of cancerous cells that mimic unicellular organisms at the very beginning of evolution [59]. Therefore, the strong neuronal–cancerous cell interaction mentioned by many investigators is probably the result of the profound dynamic changes that occur in the tumor microenvironment i.e., cellular de-differentiation, destruction of natural compartmentalization, etc.

Introducing our prototype experimental model, which is able to manipulate target tissue innervation, we previously performed a large number of experiments covering a broad spectrum of surgical biology and succeeded to explore the significant modulation of autonomic innervation in wound healing, wound contraction, inflammation, and to document the R&P denervation in the microsurgical flaps, both in rabbits and rats [40,41] (Figure 4), as well as in skin allograft transplantation (unpublished data).

In our studies, it was clear that the autonomic nervous system actively participates in regulating normal function and homeostasis, but its “organic” role is vastly enhanced after injury. This immense reinforcement of its role, we speculate, is adopted after any kind of biological stress, like injury and disease, including cancer, probably mimicking the neuron’s evolutionary/ontogenetic path [60,61,62].

### 4.1. Why Wound Healing in Cancerous Tumors Is Always Triggered, but Never Completed?

Regarding cancer, our working hypothesis states that the severe dysregulation of healing seen in malignant tumors [50] is probably due to the fact that the healing process is not activated on time during neoplastic transformation, known as the pre-tumoral or non-inflammatory phase. This most likely happens because malignant transformation is an intracellular event, and thus lacks the necessary destruction of tissue compartmentalization that would activate the healing mechanisms. In this ‘thermodynamically silent’ intracellular microenvironment, the transformed cancer cells cannot be discriminated as “foreign invaders” and are likely treated as part of the host. Healing is probably activated much later, when the normal tissue architecture is affected due to the degradation of the basement membranes by the migrating cancerous cells.

This is not contradictory to the proposal of Harold Dvorak (1986) [63], which supports that tumors are wounds that do not heal, as tumors co-opt the wound healing response in order to induce the tumor stroma required for their maintenance and growth. This supports our hypothesis of the extent to which tumors depend on their underlying substrate tissues to sustain their continued existence within the organism. The entrapment of the tumor into the radically denervated field, induced by the method presented herein, shifts the balance and makes it difficult for the tumor mass to access the much-needed extracellular space and basement membranes to progress further.

If this is the case, the critically delayed onset of wound healing in the early beginning could cause its severe dysregulation and may explain why wound healing in tumors is perpetually triggered (excessive proliferation), but never completed (no differentiation). It was macroscopically obvious in our experiments that after treatment, tumor mass shrinkage occurred due to the evident and prompt reduction in cancerous cell proliferation in the outer stromal area, while the central necrotic core continued to heal, following the classical wound contraction pattern.

The theoretical framework of our proposal may provide an explanation of the puzzling historical discovery of the Nobel laureate F. MacFarlane Burnet, the founder of immune surveillance theory, who confirmed the existence of tumor-specific antigens by showing that tumors transplanted onto syngeneic hosts are rejected, while upon transplantation, normal tissues taken from the same donor are successfully accepted [64]. It is proposed here that antigen presentation and other immune functions are closely associated with the healing process; therefore, tumors survive on the donor despite the enormous number of tumor-specific neo-antigens because the healing process was not initiated on time, thus allowing tumor cells to escape ‘host defense’, and thus enhancing tolerance, instead of immunity. Tumors are rejected when transplanted onto genetically identical recipients, and this is probably because the wound healing response and its associated immune components, including antigen presentation, is activated as a consequence of the traumatic transplantation procedure.

Indeed, based on the anticipated enhanced role of nerves in solid tumor biology, and probably due to their immense dedifferentiation, it was reasonable to expect an anticancer effect by implementing R&P denervation.

### 4.2. Nerves: The Key Healing Promoters, Do They Do the Same in Tumors?

Solid tumor innervation has been a controversial issue for many decades, but it is now well-established that sympathetic, parasympathetic, and sensory nerve fibers innervate tumors. The positive role of nerves in wound healing is an old concept [65,66] and has been shown in many recent studies [41,67,68,69,70,71]. Similarly, the existence of a firm neuron–cancer cell interaction simulating a symbiotic relationship (from Greek symbiosis = living together) has been proposed as it provides a growth advantage for both [18]. The terms neo-neurogenesis [18] and neurogenesis have been coined to describe this functional association of the nervous system and cancer [72]. A striking new finding revealed the direct metabolic support of neural terminal axons on pancreatic cancer cells, evident in the modulation of protein synthesis via mRNA translation [73]. Neurogenesis, perineural invasion, and neurotropism [74] are outstanding paradigms evidencing an active neuronal–cancer cell interaction.

It has been shown that infiltrating nerve fibers stimulate tumor growth and spreading, and reciprocally, tumor cells drive nerve outgrowth in a cross-talk that contributes to tumor progression, and it was proposed that this neuronal–cancer cross-talk goes far beyond the concept of perineural invasion and pain [19,42,75]. In addition, recent reviews provide substantial evidence that nerves are not bystanders in cancer disease [22,23,42,76], but are active participants in tumor growth and progression [10,77], and an ongoing number of investigators state clearly that the intervention on the neuro–tumoral cross-talk would be rather useful, with potential therapeutic implications [20,30,78,79,80,81,82,83,84]. Moreover, the contribution of sympathetic innervation to the formation of distant metastatic foci in breast cancer has been highlighted, and the remarkable ability of adrenergic signaling to modulate bone vasculature, driving breast cancer cell engraftment in the bone niche, has been addressed [85].

Notably, although cancer cells express many neoantigens due to numerous genetic and epigenetic changes, established tumors primarily induce immune tolerance, rather than antitumor immunity, by evolving several strategies to evade host defense. It has been shown that the host is not ignorant of the developing tumor, and this is evident from the tumor microenvironment infiltration of immune and other inflammatory cells, which is considered to be the host’s attempt to detect and eliminate emerging tumor cells [86]. However, the immune cells in the tumor microenvironment not only fail to exert potent antitumor effector functions, but they promote tumor growth [87]. An important recent discovery has shown that human cancer stem cells can produce neurons that subsequently support tumor progression [88]. Therefore, the implementation of the proposed R&P tumor denervation, which thoroughly and unconditionally interrupts the neuro–tumoral interdependence, is aligned with the impetus of those and many other similar studies.

### 4.3. Why Nerves Cannot Grow Back into Microsurgically Treated Tumors?

Autonomic nerves react immediately after injury through the collateral sprouting of the undamaged postganglionic fibers, which grow rapidly toward the original targets [89]. Collateral sprouting is considered one of the most powerful mechanisms in nature, and its universality has been shown in numerous studies. The critical prerequisite for collateral nerve sprouting is the intimate contact of their axons with the basement membranes. The disruption of this route by collagen-producing cells during wound-healing create an interfering barrier, known as scar-tissue [40]. We have demonstrated that adrenergic nerves cannot grow out in the presence of scarring and fibrosis [90], and this finding has also been noticed by others [41,91,92].

Microsurgical flap, known as free flap, is a procedure where any type of tissue can be disconnected from its supplying vessels and accompanied nerve, and transferred anywhere in the body via vascular and neural reconnection. We have also reported that the microsurgical flaps were completely denervated in the rats [93] and rabbits [90] for the time examined, which was up to 30 days (Figure 4). Another long-term study has shown that rat skin microsurgical flaps remain completely denervated at 4 wk., and even after 24 wk., the reinnervation was patchy and many arteries remained denervated [91]. Similar findings have been published by others [92,93,94]. The anastomotic site seems to be the main barrier as adrenergic nerves are not detected distally even after 30 days, as we have previously shown [90]. In the rats, only some nerves were found at 10 wk. post-op [92], and these findings have been confirmed by others [95,96].

The main reason for this «inherent» reinnervation resistance in microsurgical flaps could be the complete lack of ischemia inside the flap due to the immediate and efficient restoration of blood flow [97,98,99]. This probably precludes the massive ingrowth of neo-angiogenesis, which precedes axonal spouting in any skin flap surgery [94], a finding which mimics the embryonic/developmental order [100]. This may explain the similar results shown in microsurgical group 2 with or without the protective membrane, despite the direct contact of the tumor complex with the underlying bed in the latter. (Figure 10f–i).

### 4.4. Could Blood Flow Changes Be Responsible for Primary Tumor Regression?

Blood flow increases in both free skin [97,101,102] and free muscle flaps [103,104] after radical denervation due to the decline in vascular resistance, leading to arteriolar and capillary bed vasodilation. In comparison, the hemodynamic changes observed in the vascular pedicled flaps were found to be surprisingly similar [105,106]. These minor differences in the blood flow circulation cannot account for the dramatic differences manifested in the two hemodynamically very similar groups, microsurgical group 2 and neurovascular group 4. Nonetheless, the tumor volume steadily decreased until its elimination in the former groups, while an uninterrupted increase was shown in the latter (Figure 6a), and the LTS rate and MS time showed statistically significant (*p* < 0.001) variations (87.5% vs. 12.5% and 46.6 vs. 14.6, Figure 11c,d). In fact, we are not aware of any study showing a long-term anticancer effect through blood flow manipulation.

A significant theoretical step to advance our hypothesis was the realization of the unique opportunity provided by microvascular surgery to successfully uncouple the blood flow and innervation. Τheir coexistence in the same structure makes their separation through conventional means impossible and, in essence, resembles Siamese embryos, where the separation of one leads to the death of the other. Perhaps this close relationship was one of the reasons why the effect of innervation on the target tissues, including cancer, escaped attention for many centuries, and all of the effects were attributed exclusively to blood flow.

### 4.5. Radical and Persistent Denervation or Denervation Alone?

The neural–cancer cell interaction is far more evident in studies where denervation techniques have been applied with the aim of controlling tumor growth. Targeted tissue denervation, although it seems conceptually simple, requires a deep understanding of the dynamic and compensatory changes that occur in the peripheral nerves after injury or disease. Numerous preclinical studies have shown some anticancer effects using surgical [12,13,14,15,17,29,30,81,107,108,109,110] or chemical neural ablation [29,34,111,112,113,114], but the results were insufficient and inconsistent. Curiously, in some studies, denervation promoted tumor growth [11,16,38,115,116], probably due to the unclear segregation between partial and radical denervation and the underestimation of the enormous neural plasticity in response to trauma; this may explain why autonomic nerves have almost been ignored and were considered as passive bystanders in cancer disease for so many centuries.

The present approach may provide an explanation of why the transection of the trigeminal nerve and its accompanying vessels, attempted two centuries ago, induces only a temporal reduction in the ulceration, followed by tumor recurrence, in all cases [117]. It may also explain why tumor denervation has been reported to promote tumor growth and to enhance cancer metastasis [81,118]. Moreover, gastrectomy combined with vagotomy in rats induced gastric mucosa denervation, leading to an increase in tumor development (67%) [17]. This high rate of recurrence can be attributed to the maintenance of natural tissue continuity, which greatly facilitates reinnervation from neighboring supplementary sources, and most importantly, because the sympathetic and sensory components were left intact.

Our results have also shown that minimizing the future reinnervation potential is an essential part of the denervation therapeutic strategy. Compared to microsurgical group 2, the omission of the anastomosis in vascular group 3 sharply increased the morbidity and mortality, followed by a new increase in both parameters after nerve retention in neurovascular group 4 (Figure 12). They also indicate that there is no need for the R&P denervation to be long-lasting, but it seems that a short ‘denervation window’ is efficient to induce an effective systemic treatment. It is generally believed that even if one cancer cell is left behind, it may become a new tumor; this further strengthens the need for radicality.

### 4.6. Are Microsurgical Transplants the Manifestation of Neural-Target Interdependence?

Since the 1970s, microsurgical flaps have revolutionized the reconstitution of complex post-traumatic injuries by transferring large tissue segments in a single surgical step. Close examination reveals an ‘inherent’ resistance to reinnervation, and this has limited their use in functional reconstructive microsurgery, despite the initial enthusiasm. Poor functional results have been reported for microsurgical skin or myocutaneous [119,120], muscle [121,122], and bone transplants [123], and this dysfunction is reversed if regional muscle units with an intact neurovascular pedicle are used [124,125]. Tissue renewal and homeostasis are similarly impaired in skin free flaps, made evident by trophic ulcers, even if the accompanying nerves are sutured and re-connected [40,41].

Similar observations were made during our introductory studies, where extensive skin ulcers in the microsurgical, but not in the vascular pedicle, flaps were observed (unpublished data, Figure 4). Severe contraction was shown in the rabbit microsurgical flaps, by up to 50%, as early as day 30 (Figure 4a,b). This was associated with excessive scarring and fibrosis [90], whereas the pedicle flaps displayed light contraction (Figure 4c,d). Similarly, hair growth, the physical sign of regeneration, was extremely poor inside the microsurgical flaps (Figure 4b); in stark contrast, superabundant hair growth inside the vascular pedicle flaps was observed (Figure 4c).

Another important finding was the absence of immunological responsiveness in the microsurgical flaps, which was present in the conventional skin autografts, indicating that the healing pattern is substantially different [126]. This was further confirmed by the lack of skin allograft rejection in the rabbits when transplanted onto the surface of the radically denervated microsurgical flaps, followed by an extraordinary acute hyper-eosinophilia of >60% (normal values 0–6%), a condition which is usually associated with allergic and autoimmune reactions or graft versus host disease. In stark contrast, the partially denervated vascular flaps did reject the skin allografts (unpublished data).

Taken together, if R&P denervation provokes such detrimental effects on tissue homeostasis and functioning, the fact that tumors cannot survive in this environment should not be surprising, but rather, is an expected outcome.

### 4.7. Can a Tumor Entrapped into a Microsurgical Flap Eventually Survive?

We have shown here that the microsurgical tumor complex has shown superiority in subject survival by inducing primary tumor regression and increased LTS rates and MS times. A great difference in the tumor volume curves between microsurgical group 2 and neurovascular group 4 was also recorded (Figure 10a), and a significant difference (*p* < 0.002) in the LTS rate between the same groups has also been recorded (90% vs. 12.5%, Figure 6c).

Of particular interest is the comparison of microsurgical group 2 with vascular group 3, in which all apparent neural structures were severed and the only connection with the host was the tiny artery and vein. Therefore, the omission of microvascular anastomosis alone caused a disproportionate increase in mortality and morbidity, evidenced by the significant decline (*p* < 0.001) in the LTS rate and MS time (90% vs. 40% and 47.7 vs. 25.8 wk., respectively, Figure 11a,b). This is a compelling finding because it highlights the importance of microvascular anastomosis, per se, as the possible key therapeutic step (Figure 11c,d). It has been shown that reinnervation in vascular flaps takes place via the intact vascular pedicle, where a slightly denser distribution of nerve fibers and bundles compared to the control tissue and hypertrophic nerve sprouts centered around the blood vessels were observed, even when the accompanying nerve was severed [94].

It therefore seems that R&P denervation probably mediates the aforementioned findings. This is more evident after the omission of microvascular anastomoses per se, which reversed these biological effects in vascular group 3 and in neurovascular group 4, which are roughly identical surgical preparations in terms of the trauma severity (Figure 11). To the best of our knowledge, microsurgical auto-transplants—although they are highly demanding preparations and carry a certain but small risk for total necrosis due to anastomotic failure—are the most appropriate and effective surgical methods for the complete and sustained separation of a viable tissue area from its natural innervation to date.

It can be hypothesized that microsurgical preparation converts the tumor complex into a ‘black hole’ for the rest of the body, in the sense that no harmful signals against the host can escape anymore from the malignant tumor, thus giving to the host’s defense the opportunity to prevail by shifting the balance in its favor.

### 4.8. Does Neuro-Immune Interplay Leverage Cancer Progression and Metastasis?

In addition to the direct neuro–tumoral effect, the nervous system may exert its influence on the tumor biology through the immune system. It has been shown that T-cell-derived acetylcholine can inhibit Tumor Necrosis Factor production [127] and can bind to the nicotinic and muscarinic receptors on lung cancer cells to accelerate their proliferation, migration, and invasion [128]. Nerves in the tumor niche are in cross-talk with the immune system, which could contribute to tumor progression via inflammation [129]. Moreover, it has been reported that sympathetic activation directly promotes metastasis by stimulating macrophage infiltration, inflammation, angiogenesis, etc., by inhibiting the cellular immune responses and programmed cell death [22]; in addition, the sympathetic and parasympathetic density and function have an opposing effect on the expression of certain immune checkpoint molecules and on the clinical outcomes in breast cancer patients [130].

### 4.9. Abscopal Effect Induction: Can We Turn Cancer against Itself?

The strong anticancer effect transmitted to remote, non-treated tumors shares similar features with the well-known phenomenon in oncology [131] of the abscopal effect, or bystander response, meaning off-target, and has been defined as the ability of localized cancer treatment to trigger systemic antitumor effects. The abscopal effect was initially observed when targeted irradiation applied on a primary or metastatic tumor caused the shrinking of the treated tumor, as well as the shrinking of the tumors outside the scope of the localized treatment. Many animal studies have used bilateral tumors to monitor the abscopal effect [132,133,134,135,136,137,138,139,140,141], and its reliability is based on the fact that all implanted tumors share an identical phenotypic profile, like the naturally occurring distal metastatic lesions. The abscopal effect is an extremely rare phenomenon, accounting for about one case per year, globally. The immune system has been pinpointed as the key component [142], but the exact mechanism is still unclear, thus preventing efficient reproduction. The published clinical data, although promising [135,143,144,145], does not yet facilitate its routine implementation in clinical practice. The very interesting point is that it works equally well in both directions, from a primary tumor to metastatic lesions, and vice versa [146].

In the present study, we have shown the possibility of purposefully and reliably inducing the abscopal, or similar, anticancer effect in metastatic tumors at a high rate (57.1%) without exogenous treatment. It is assumed that the significant difference in our results relies on the effectiveness and depth of the R&P therapeutic denervation induced on the primary tumor. This is supported by a recent article presenting the conclusion that radiation therapy of peripheral glioma tumors consistently transmitted a tumor-specific immune response in the intracerebral tumors only when peripheral tumors were successfully cured by irradiation [146].

### 4.10. Do Nerves Interfere with the Abscopal Response, at All?

The interaction of the peripheral nervous system and cancer raises the question of the potential involvement of the former in the abscopal response. There are some reasons to suspect that the nervous system might be implicated in this phenomenon. It has been documented that the abscopal response is exclusively associated with an independent local mechanism [147,148]. In many instances, abscopal responses in normal tissues involve the contralateral site with a precision referring to the anatomical distribution of the peripheral nerves [149]. A dramatic increase in contralateral lung abnormalities (DNA damage, cellular infiltration, etc.) following ipsilateral lung irradiation in breast cancer patients, even in shielded lung segments, has been reported [150]. Radiation oncology, the dominant activator of the abscopal effect, damages the peripheral nerves, which are more sensitive to large fractions of radiation, and this effect is long-lasting [145].

Furthermore, the median duration of the abscopal response in patients takes about 21 months to develop [151], and once an abscopal response was achieved, a median time of 13 months went by before disease progression occurred [152]; this long time-frame can be associated more with neural, rather than to immunological, mechanisms. In fact, this view has gained direct support from a very new report, showing the implication of the adrenergic function in human abscopal responses; the frequency of abscopal events following radiation treatment alone is highly dependent upon the degree of adrenergic stress in the host, and these data reveal an unexpected degree of control of the sympathetic nervous system on both the irradiated and non-irradiated distant tumor sites [152].

## 5. Conclusions and Perspectives

The induction of a potent local and global anticancer response in an immunocompetent host, effected through R&P therapeutic denervation applied on the primary tumor “in situ”, followed by its systemic transmission, leading to a high rate of long-term (>1 year) survival, was presented herein. The local to global therapeutic conversion was an extremely pleasant surprise, which exceeded our original prediction.

Supposing that this study may raise more scientific questions than the answers it offers, our approach is distinct because it has shown that:

- Tumor denervation must be radical (100%), as well as persistent, otherwise treatment failure will occur.
- Local and global cancer treatment can be induced without tumor removal.
- Effective local treatment is a prerequisite for the triggering and propagation of the abscopal effect with an anticipated rate, strength, and reproducibility.
- If denervation is prompt and radical, there is no need for it to be permanent, but a short “denervation window” might be sufficient to induce an effective global treatment.
- Cancer treatment may be achieved without systemic toxicity, and in a fertility-preserving way.
- It provides direct evidence in vivo and consolidates the firm neuro–tumoral cross-talk, as has been speculated by many scientists for many centuries.

Despite the very promising results, we should acknowledge some limitations. The experiments with the animals were very laborious and intensive. Although there were several animals per group, the number of groups was too large to allow more animals to be examined per experimental group. Important evidence on the exact mechanism still needs to be collected, with detailed neurohistochemical studies on the changes occurring in the tumor microenvironment in the various surgical manipulations. Also, the extrapolation of the results to other types of cancerous lesions in different preclinical models would be very helpful. Finally, as the microsurgical therapeutic denervation presented here can be tested on human cancer patients using the primary tumor or any distal metastatic focus, results from first in-human clinical trials are needed.

Based on these findings, a proof-of-concept clinical trial has been approved by the Scientific Advisory Board and the Ethical Committee of University Hospital of the University of Patras, Greece, and is currently ongoing (Ref. no: 28497/2.11.18); this will provide more evidence regarding the clinical value of this innovative approach.

## Data Availability

Data sharing not applicable.
